# Why does the adverse effect of inappropriate MRI for LBP vary by geographic location? An exploratory analysis

**DOI:** 10.1186/s12891-019-2964-7

**Published:** 2019-11-30

**Authors:** Mujahed Shraim, Manuel Cifuentes, Joanna L. Willetts, Helen R. Marucci-Wellman, Glenn Pransky

**Affiliations:** 1 Department of Public Health, College of Health Sciences, QU Health, Qatar University, P.O.Box: 2713, Doha, Qatar; 2 Regis College, 235 Wellesley St, Weston, MA USA; 3 Liberty Mutual Research Institute for Safety, Hopkinton, MA USA; 4 The Workers Compensation Research Institute, Cambridge, MA USA; 5 University of Massachusetts Medical School, 55 N Lake Ave, Worcester, MA USA

**Keywords:** Cohort study, Low back pain, Work disability, Geographic variation in care, Magnetic resonance imaging

## Abstract

**Background:**

Early magnetic resonance imaging (eMRI) for nonspecific low back pain (LBP) not adherent to clinical guidelines is linked with prolonged work disability. Although the prevalence of eMRI for occupational LBP varies substantially among states, it is unknown whether the risk of prolonged disability associated with eMRI varies according to individual and area-level characteristics. The aim was to explore whether the known risk of increased length of disability (LOD) associated with eMRI scanning not adherent to guidelines for occupational LBP varies according to patient and area-level characteristics, and the potential reasons for any observed variations.

**Methods:**

A retrospective cohort of 59,360 LBP cases from 49 states, filed between 2002 and 2008, and examined LOD as the outcome. LBP cases with at least 1 day of work disability were identified by reviewing indemnity service records and medical bills using a comprehensive list of codes from the International Classification of Diseases, Ninth Edition (ICD-9) indicating LBP or nonspecific back pain, excluding medically complicated cases.

**Results:**

We found significant between-state variations in the negative impact of eMRI on LOD ranging from 3.4 days in Tennessee to 14.8 days in New Hampshire. Higher negative impact of eMRI on LOD was mainly associated with female gender, state workers’ compensation (WC) policy not limiting initial treating provider choice, higher state orthopedic surgeon density, and lower state MRI facility density.

**Conclusion:**

State WC policies regulating selection of healthcare provider and structural factors affecting quality of medical care modify the impact of eMRI not adherent to guidelines. Targeted healthcare and work disability prevention interventions may improve work disability outcomes in patients with occupational LBP.

## Background

Occupational Low back pain (LBP), defined as reported pain in the lumbar region as the primary reason for medical visit and registered as such (occupational origin) in the clinical records, is very common and accounts for a third of work-related soft and hard tissue musculoskeletal injuries and disorders leading to work absenteeism [1], and is the leading cause of years lived with disability globally [2]. Due to its use in Workers’ Compensation, we will refer to low back injury or disorder as an injury and by doing so will use the expression “injured workers.” Although the majority of workers (68%) with LBP resulting in work absence return to work within few weeks, a significant proportion of occupational LBP cases experience prolonged work disability [3], which is associated with higher risk of permanent disability [4]. As many persons affected by LBP are of working age, work disability is a key outcome in LBP. Prolonged work disability is associated with significant health and economic impacts, and is a priority outcome in evaluation of treatment effectiveness [5].

Prior studies have shown that length of disability (LOD) due to occupational LBP is associated with several factors. These include, individual characteristics (such as age, gender, tenure), physical demand of job and employer/work environment related characteristics [6, 7], regional factors such as state workers’ compensation (WC) policies [8] and residential area socioeconomic characteristics [9], and health care-related factors, primarily reflecting treatments that are inconsistent with accepted clinical guidelines. These include early opioid prescribing (within the first 15 days of seeking medical care), early magnetic resonance imaging (eMRI) scanning not adherent to evidence-based clinical guidelines (within the first 30 days of first registered medical visit for the current low back pain episode), prolonged or passive physical therapy, and other interventions not recommended by evidence-based clinical guidelines [10–13].

Clinical practice guidelines for acute nonspecific LBP recommend that, except for suspected serious underlying conditions (e.g. cancer and infection), MRI scanning should not be performed until at least a one-month period of standard medical therapies has occurred, and is only then indicated to evaluate patients with persistent LBP and radiculopathy or spinal stenosis who may be candidates for surgery [10]. Despite these guidelines, inappropriate eMRI in patients with acute LBP is common, and is associated with prolonged disability, unnecessary subsequent interventions, and higher medical costs [14–16]. In addition, this practice has been resistant to various efforts to curtail it. However, whether its negative impact is the same across all cases is unknown. Some forms of ineffective or inappropriate care have differential impacts on different populations. For example, low back surgery is associated with worse disability outcomes for WC cases than non-WC cases [17]. Information on relative impact can be helpful to prioritize interventions for groups who might be most adversely affected by a certain practice or risk factor, or regions where a risk factor has higher impact on outcomes. Although there are significant geographic variations in the prevalence of eMRI scanning for LBP [18, 19], whether the risk of increased LOD associated with eMRI scanning for occupational LBP varies is unknown.

The aim of this exploratory study was to examine whether the magnitude of increase in LOD associated with eMRI scanning for occupational LBP varies according to a range of different factors, as a way of identifying potentially susceptible subpopulations, and thus providing new information on effect modification, and guidance for prioritizing interventions to decrease this practice. A large national database of injured workers provided a unique opportunity to examine individual, local and state factors that might affect susceptibility.

## Methods

### Study population

This was a retrospective cohort of LBP cases identified from the administrative database of a large WC private company, which accounts for about 10% of the WC coverage in the United States [20]. Workers compensation is a no-fault, compulsory, employer-paid insurance system that provides coverage for medical care and a percentage of lost wages for workers who have an injury caused or substantially aggravated by work. Each State has a slightly different system, based on specific state laws. The insurer pays all bills for medical care regardless of provider, so the record of medical care is quite complete. Medical bills are required to include diagnoses, date and type of service, and provider.

The database includes comprehensive information about medical care and work disability compensation received by injuried workers. The distribution of occupational injuries from the dataset is similar to other large national work injury databases [21], and our dataset has been used to conduct several national occupational research studies [15, 22, 23]. We included all LBP cases filed between 2002 and 2008 (inclusive), aged 18–65 years at first occupational LBP registered visit and received disability payment for at least 1 day. The New England Institutional Review Board approved the study.

Briefly, International Classification of Diseases, Ninth Edition (ICD-9) codes referring to LBP or nonspecific back pain were used to identify uncomplicated LBP patients by reviewing the primary physician reported ICD-9 codes in medical bills for services during the first 15 days of seeking medical care (Additional file 1: Table S1).These diagnoses are meant to describe the injury and any other relevant diagnoses. In prior investigations, we found that almost all of the relevant diagnoses were represented in these bills, which could each contain up to five different diagnoses. We looked at all diagnoses in the first 15 days, and included only those cases where LBP represented at least 66% of all musculoskeletal diagnoses (ICD 320–399, 710–739, and 800–999). The majority of cases (89%) had 100% of their ICD-9 diagnoses representing low back pain. Complicated LBP cases with specific ICD-9 codes indicating severe injuries, multiple trauma, or significant non-injury diagnosis (e.g., cancer, autoimmune disease, or infection) were excluded.

### Data and measures

#### Outcome variable

The outcome was LOD calculated as total number of lost workdays from the beginning to the of wage replacement payment. We censored LOD at 1 year from the start of work disability because 93% of LBP cases return to work within 1 year, and to prevent the influence of state WC policies regarding termination of long-term disability claims with lump-sum settlements that often happen after a year of work disability duration [24].

#### Predictor variables

##### Individual-level variables

We included individual-level variables that have been reported as significant predictors of LOD in patients with occupational LBP [8]. These include age, sex, job tenure, average weekly wage, industry type, severity of LBP injury, lumbar spine surgery, early opioid prescribing (within 15 days of injury), eMRI, and WC claim litigation status. Operational definitions of these variables are described in detail elsewhere [8]. Briefly, early opioid prescribing was identified by reviewing medical bills, eMRI and lumbar spine surgery status were identified using Clinical Procedural Terminology codes [25]. eMRI was identified as a gap of 30 days or less between the date of first treatment for the low back injury and the date of the first lumbar MRI procedure. This was based on coexisting ICD-9 codes and Clinical Procedural Terminology codes reported in medical bills.

##### Neighborhood-level variables

We included “neighborhood” inflation-adjusted median household income (MHI), quantified at census-tract level, which was found to be associated with LOD in cases with occupational LBP [9].

##### State-level variables

We included several state-level variables found to be associated with healthcare utilization and work disability outcomes in patients with LBP and other conditions [26, 27]. These included the annual state physician density per 100,000 population (2002–2008) [28] and state orthopedic surgeon density per 100,000 population. State orthopedic surgeon density was available only for 2004 and 2005 [29, 30]. Therefore, data on state orthopedic surgeon density from 2004 were used as an estimated density for 2002–2004 and data from 2005 were used for 2005–2008. State MRI facility density per 100,000 population was another variable of interest. Prior studies have reported significant variation between states in eMRI for LBP, but it is not clear if MRI facility density is independently related to LOD. Other variables included were state annual unemployment rate and annual state WC policies on wage replacement and medical benefits (2002 to 2008), which are associated with LOD in patients with occupational LBP [8]. Neighborhood-level and state-level data were obtained from national and private data sources; see Additional file 2: Table S2.

### Data analysis

We used descriptive statistics to summarize included variables. We used multivariable multilevel regression analysis to model our hierarchical data (LBP patients nested within different states) using the PROC MIXED function in SAS 9.2 [31]. The distribution of LOD was positively skewed. Therefore, the natural logarithm of LOD and log-linear models were used in analysis. Continuous predictor variables were grand-mean centered. The majority of census tracts (96%) had less than five LBP cases. Therefore, neighborhood MHI was included in all analyses as individual-level variable to avoid less efficient estimate of variance parameters using small cluster sizes [32].

We followed a stepwise modelling approach to assess if the effect of eMRI scanning on LOD varies significantly between states and whether such variation is moderated by included individual and state-level predictors. Briefly, the first model (empty model) estimated mean LOD across all included states and estimated the amount of variability in LOD attributed to state-level factors using the intraclass correlation coefficient (ICC) [8, 33]. Model 2 included all state-level variables to identify independent predictors of LOD. Model 3 included statistically significant predictors of LOD from model 2 plus all individual level variables as fixed effects to identify individual level predictors of LOD. Model 4 included all significant predictors of LOD identified in model 3 plus eMRI as random effect variable to assess if the independent effect of eMRI on LOD varied between states significantly. Model 5 included significant predictors from model 4 plus within and across-level interactions to examine if the effect of eMRI scanning on LOD was moderated by other individual and state-level variables.

## Results

A total of 59,360 LBP claimants from 48 states and the District of Columbia were included. Two states (North Dakota and Wyoming) had very few LBP claims, and thus, were excluded. The average LOD (censored at 1 year) was 98 days (median = 43 days); more detailed summary of distribution of LOD by state is given elsewhere [8]. About 69% of cases were men. The mean age and tenure of LBP claimants were 39.4 years (standard deviation (SD) = 10.8) and 5.8 years (SD = 7.7), respectively. A total of 17,555 cases (29.6%) received eMRI scanning for LBP. A summary of the cohort characteristics is given in Table 1. The estimated ICC showed that 5% of between-state variability in mean LOD is explained by state-level characteristics. As shown in Table 2 (model 5), state WC policy variables, state orthopaedic surgeons density, state MRI facility rate, and cross-level interactions with eMRI scanning explained 65% of between-state variability in mean LOD.

**Table 1:** Cohort characteristics according to individual and state-level variables

Variable	Number	Percentage	Mean (minimum, maximum)	SD
Gender				
Female	18,352	30.9		
Male	41,008	69.1		
Age (years)			39.4	10.8
Tenure (years)			5.8	7.7
Average Weekly Wage ($)			403.0	187.5
Injury Severity				
Less severe	47,805	80.5		
More severe	11,555	19.5		
Early opioid prescribing (MEA per 100 mg)				
No	43,013	72.5		
Yes	16,347	27.5	3.9	3.8
Early lumbar MRI scan				
No	41,805	70.4		
Yes	17,555	29.6		
Lumbar spine surgery				
No	53,869	90.7		
Yes	5491	9.3		
Industry type				
Mining	1071	1.8		
Construction	2087	3.5		
Transportation, Communications, Electric, Gas, and Sanitary Services	16,305	27.5		
Agriculture, Forestry, and Fishing	588	1.0		
Manufacturing	10,887	18.3		
Wholesale Trade	4515	7.6		
Retail Trade	6626	11.2		
Services	14,782	24.9		
Public Administration	1990	3.4		
Finance, Insurance, and Real Estate	509	0.9		
Litigation status				
Yes	19,182	32.3		
No	40,178	67.7		
Median household income ($K)			52.7	21,394
Wage replacement rate (%)			68	3
Waiting period (days)			5.3	1.9
Retroactive period (days)			15.7	7.0
State medical fee schedule				
No	10,498	17.7		
Yes	48,862	82.3		
Initial treating provider choice				
Allowed	22,516	37.9		
Not allowed	36,844	62.1		
Treating provider change				
Allowed	8282	14.0		
Allowed once	14,241	24.0		
Not Allowed	36,837	62.1		
Annual unemployment rate			5.3	1.0
Annual number of state active Orthopedic surgeons per 100,000 population in 2004 and 2006			6.3	0.99
Annual number of state active physicians per 100,000 population			272.1	66.6
Number of state MRI facilities per 100,000 population in 2006			2.4	0.62

*SD* Standard deviation, *MRI* Magnetic resonance imaging

**Table 2:** Parameter estimates from the five multilevel regression models examining the associations of LOD with individual-level and state-level variables

Parameter	Length of disability				
	Model 1	Model 2	Model 3	Model 4	Model 5
Intercept	3.835***	3.880***	3.268***	3.269***	3.346***
Gender					
Female			0.078***	0.075***	0.040*
Male^a^					
Age (years)			0.005***	0.005***	0.005***
Tenure (years)			−0.004***	−0.004***	−0.004***
AWW/$100			0.017***	0.013***	0.013**
Industry type					
Mining			0.452***	0.445***	0.443***
Construction			0.193***	0.183***	0.182***
Transportation			0.258***	0.247***	0.247***
Agriculture			0.090	0.083	0.081
Manufacturing			0.126**	0.118**	0.117**
Wholesale trade			0.084	0.076	0.076
Retail trade			0.127**	0.119**	0.118**
Services			0.101*	0.092*	0.091*
Public administration			0.077	0.069	0.068
Finance^a^					
Injury severity					
More severe			0.086***	0.085***	0.085***
Less severe^a^					
Early opioid/100 mg MEA			0.014***	0.014***	0.014***
eMRI scan					
Yes			0.426***	0.435***	0.402***
No^a^					
Lumbar spine surgery					
Yes			0.719***	0.715***	0.712***
No^a^					
Litigation status					
Yes			1.144***	1.152***	1.152***
No^a^					
Median household income ($)			−0.007***	−0.007***	− 0.007***
State physician density		−0.001***	−0.001*	− 0.001**	- < 0.001
State Orthopedic surgeons density		−0.027	0.070	0.070	0.042*
MRI facility rate		−0.078	−0.144***	− 0.129***	−0.182***
Wage replacement rate		0.002	0.004	0.004	0.004
Waiting period		0.052***	0.069***	0.069***	0.069***
Retroactive period		0.006**	0.006***	0.006***	0.006***
Treating provider change					
Allowed		0.234***	0.115***	0.109***	0.114***
Allowed once		0.001	−0.118**	− 0.116***	− 0.114***
Not allowed^a^					
Treating provider choice					
Allowed		0.165***	0.270***	0.261***	0.332***
Not-allowed^a^					
State medical fee schedule					
Yes		0.057*	0.100***	0.100***	0.098***
No^a^					
Unemployment rate		0.024**	0.013*	0.012*	0.011
MRI (yes) X State Orthopedic surgeons density MRI (yes) X State Orthopedic surgeons density					0.053***
MRI (yes) X Dr choice (yes) MRI (yes) X Dr choice (no)					0.099**
MRI (Yes) X male gender MRI (Yes) X female gender					−0.047**
MRI (yes) X MRI facility density MRI (no) X MRI facility density					−0.058**
Variance components					
Within-state variability	1.393***	1.390***	0.914***	0.910***	0.910***
Between-state variability	0.072***	0.035***	0.026***	0.025***	0.025***
ICC	5%	2%	3%	3%	3%
Proportional reduction in between-state variability		51%	64%	65%	65%
Random slope					
eMRI				0.011**	0.011**
Model fit statistic					
-2LL	188,306	188,221***	163,485***	163,323***	163,320*
BIC	188,310	188,229***	163,493***	163,342***	163,339*

*AWW* Average weekly wage, *MEA* Morphine equivalent amount, *eMRI* Early magnetic resonance imaging, *ICC* Intraclass correlation coefficient, *−2LL* −2 log likelihood ratio, *BIC* Bayesian information criterion

*Indicator of statistical significance *p*<. 05; **p<. 01; ***p<. 001

^a^Indicator of reference group

### Geographic variation in impact of eMRI scanning on LOD

**Fig. 1:**
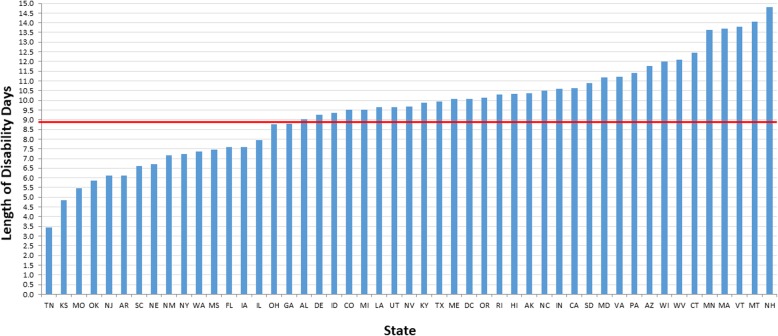
Adjusted Effect of Early MRI on Length of Disability by State

As shown in Table 2 (Regression slopes), we found statistically significant between-state variations in the negative effect of eMRI on LOD. Overall, eMRI was associated with increase in mean LOD by 9.4 days (95% CI 8.5, 10.2), and this varied across states from 3.4 days in Tennessee to 14.8 days in New Hampshire (Fig. 1).

### Associations of state-level factors with LOD

As shown in Tables 2 and 3, after controlling for statistically significant predictors and interactions, an increase in state orthopedic surgeon density by 1 surgeon for each 100,000 population was associated with an increase in mean LOD by 1.2 days (95% CI 0.2, 2.3). Additionally, an increase in the state MRI facility density per 100,000 population by 1 facility was associated with decrease in mean LOD by 4.7 days (95% CI 2.9, 6.4). There was no statically significant association between state physician density and LOD.

**Table 3:** Adjusted associations between individual-level and state-level variables with length of disability estimated by multivariable multilevel regression (model 5)

Variable	Length of disability days				
	Parameter estimate of association	S.E.	Difference in the geometric mean of LOD	95% CI	*p*-value
Intercept	3.346	0.055	28.4	25.4, 31.7	<.001
Gender					
Female	0.040	0.017	1.2	0.2,	.02
Male^a^				2.2	2
Age	0.005	< 0.001	0.1	0.1, 0.2	<.001
Tenure	−0.004	0.001	−0.1	− 0.2, − 0.1	<.001
AWW/$100	0.013	0.005	0.4	0.1, 0.6	.004
Industry					
Mining	0.443	0.053	15.8	11.5, 20.6	<.001
Construction	0.182	0.048	5.7	2.6, 9.0	<.001
Transportation	0.247	0.044	7.9	4.9, 11.2	<.001
Agriculture	0.081	0.059	2.4	−1.0, 6.2	.167
Manufacturing	0.117	0.044	3.5	0.9, 6.4	.008
Wholesale trade	0.076	0.045	2.2	−0.4, 5.1	.096
Retail trade	0.118	0.045	3.6	0.9, 6.5	.009
Services	0.091	0.044	2.7	0.1, 5.5	.038
Public administration	0.068	0.048	2.0	− 0.7, 5.0	.157
Finance^a^					
Injury severity					
More severe	0.085	0.010	2.5	1.9, 3.2	<.001
Less severe^a^					
Early Opioid/100 mg MEA	0.014	0.002	0.4	0.3, 0.5	<.001
eMRI scan					
Yes	0.402	0.022	9.4	8.5, 10.2	<.001
No^a^					
Lumbar spine surgery					
Yes	0.712	0.022	29.5	27.1, 32.1	<.001
No^a^					
Litigation status					
Yes	1.152	0.009	61.4	59.8, 63.1	<.001
No^a^					
Median household income ($)	−0.007	0.002	−0.2	−0.3, −0.1	<.001
State physician density	<−0.001	< 0.001	0.0	0.0,0.0	.063
State Orthopedic surgeons density	0.042	0.018	1.2	0.2, 2.3	.021
MRI facility rate	−0.182	0.037	−4.7	−6.4, −2.9	<.001
Wage replacement rate	0.004	0.003	0.1	0.0, 0.3	.116
Waiting Period	0.069	0.007	2.0	1.6, 2.5	<.001
Retroactive Period	0.006	0.002	0.2	0.1, 0.3	<.001
Treating provider choice					
Allowed	0.332	0.034	11.2	8.5, 14.0	<.001
Not allowed^a^					
Treating provider change					
Allowed	0.114	0.031	3.4	1.5, 5.5	<.001
Allowed once	−0.114	0.033	−3.1	−4.7, −1.4	<.001
Not allowed^a^					
State medical fee schedule					
Yes	0.098	0.020	2.9	1.7, 4.2	<.001
No^a^					
Unemployment rate	0.011	0.006	0.3	0.0, 0.7	.066
MRI (yes) X State Orthopedic surgeons density MRI (No) X State Orthopedic surgeons density^a^	0.053	0.014	1.5	0.7, 2.2	<.001
MRI (yes) X Dr choice (yes) MRI (yes) X Dr choice (no)^a^	0.099	0.030	2.7	1.1, 4.2	.002
MRI (Yes) X male gender MRI (Yes) X female gender^a^	−0.047	0.019	−1.4	−2.6, −0.2	.002
MRI (yes) X MRI facility density MRI (no) X MRI facility density^a^	−0.058	0.023	−1.7	−3.1, −0.3	.014

*S.E.* Standard error of parameter estimate of association, *CI* Confidence intervals, *LOD* Length of disability, *AWW* Average weekly wage, *eMRI* Early magnetic resonance imaging, *MEA* Morphine equivalent amount

^a^Indicator of reference group

### Within and cross-level interactions with effect of eMRI on LOD

We found statistically significant interactions between gender and effect of eMRI on LOD; the negative effect of eMRI on mean LOD was higher by 1.4 days in females than males (95% CI 0.2, 2.6; see Table 3). In addition, the negative effect of eMRI on mean LOD increased by 2.7 days (95% CI 1.1, 4.2) in LBP cases working in states that do not limit initial treating provider choice than those working in states that limit initial treating provider choice. Additionally, the effect of eMRI on mean LOD increased by 1.5 days (95% CI 0.7, 2.2) with an increase in state orthopedic surgeon density by 1 surgeon for each 100,000 population. Finally, the effect of eMRI on mean LOD was lower by 1.7 days (95% CI 0.3, 3.1) with an increase in number of state MRI facility density by 1 facility for each 100,000 population. No other statistically significant interactions were observed between effect of eMRI on LOD and the remaining individual and state-level variables.

## Discussion

To our knowledge, this is the first study to explore geographic variation in the negative impact of eMRI for occupational LBP and factors associated with such variation. Findings from the study showed significant regional variations in the negative impact of eMRI on LOD due to LBP after adjusting for between-state differences in individual-level, neighborhood-level, and state-level characteristics associated with LOD in LBP patients. The observed variations in the negative impact of eMRI on LOD were mainly explained by female gender, state WC policy not-limiting initial treating provider choice, higher state orthopedic surgeon density, and lower state MRI facility density.

The finding of more negative impact of eMRI on LOD in women can be explained by role and interpretation. For example, men might be more likely expected by their providers to have back pain at work and recover, even with a relevant clinical abnormality. Common abnormalities seen on MRI, such as disc degeneration, disc protrusions, mild arthritis, and vertebral endplate changes, are often of uncertain clinical significance, and are frequently seen in asymptomatic persons [34, 35]. However, women may be more likely to have over-interpretation of abnormalities by doctors less used to seeing women in manual labor job roles [36]. Female injured workers are also more likely to have non-localized LBP, thereby a ‘positive’ MRI might be more likely to be over-interpreted, and this also could lead to prolonged disability [37].

State WC policy not limiting initial treating provider choice is associated with increased LOD [8]. There is some evidence that workers who choose their treating provider have longer disability duration than those treated by medical provider networks chosen by employers [38, 39]. Therefore, the negative impact of eMRI on LOD in the context of unlimited provider choice may mean that eMRIs are less justified and perhaps more often over-interpreted than those done by doctors with more occupational LBP experience.

The negative impact of eMRI on LOD in the context of higher state orthopedic surgeon density may be explained by more demand for patients or excess orthopedic surgical capacity, and thus more likely over-interpretation of significance of findings, which may result in more diagnostic labelling of patients, increasing the sick role, and unnecessary interventions. For example, higher state orthopedic surgeon density has been independently linked to higher back surgery rates in workers with LBP [26].

The finding about the negative impact of eMRI on LOD with lower state MRI facility density may be explained by variations in interpretation of eMRI findings and prevalence of interpretive errors, which might be associated with type of equipment and imaging sequence used in MRI facilities, availability of fellowship-trained radiologists, and expertise in interpreting MRIs or nuances in reporting results in facilities performing a high number of scans [40, 41].

This study adds an important contribution to current knowledge in occupational LBP by showing that an important risk factor for adverse outcome (eMRI for LBP not adherent to evidence-based clinical guidelines) can vary in impact, and identifies the personal and local factors associated with this variation. Understanding the basis of this variation in disability outcomes is key to focus efforts to improve healthcare and work disability outcomes for LBP patients. A unique strength of this study is that the observed associations were independent of several important predictors of work disability in LBP, including individual-level variables (e.g. age, early opioid prescribing, back surgery, etc.), neighborhood MHI, and state-level characteristics (WC policies and annual unemployment rate) identified through current literature and our explanatory models. Another important strength is that our study included a large national sample of occupational LBP cases, filed over a period of 7 years in 49 states, which are representative of private industry workers. This sample has similar distribution of demographic characteristics reported in prior studies examining occupational LBP [42–44], and national occupational datasets with respect to distribution of occupational injuries and associated medical costs [21]. Additionally, our dataset has comprehensive information on medical and indemnity services, which enabled us identify LBP cases using a list of specific and standardized ICD-9 codes.

Currently, the mechanism linking the predictor eMRI with health and financial low back pain outcomes is not clear. Some potential reasons are that not following guidelines with one procedure maybe a marker for similar prescribing process with further procedures. More dynamics interpretations point to affecting patients’ decision process by feeding their concern and anxiety, which results in requesting from their providers treatments that are more intensive and in a speedy manner [45, 46]. All of this would cause patients who reinforce their idea of having a serious disease, pressured providers willing to prescribe procedures and sick-leave with the intention to assure patients they are being taken care of. Further research is needed to clarify these mechanisms, which may shed light on potential plausible interventions to improve health outcomes in patients with LBP.

This study also has other limitations. One of them is that LOD was measured using wage replacement data, but termination of wage replacement benefits does not necessarily indicate recovery and return to work, which may underestimate LOD. Additionally, WC administrative data lack information about injury severity and level of functional disability. However, we accounted for low back injury severity (more severe, less severe) using a validated list of ICD-9 codes used in prior studies [8, 9, 47]. In the current study, low back injury severity was a significant predictor of LOD. Additionally, a longer period between the beginning of symptoms and the first visit may be represent either or both, a mild pain that extends for a long period or a mild pain that gets worse. It is possible that some patients who received an eMRI had had a longer period with LBP and, therefore, met the criteria to have an MRI. The extent of this potential misclassification is unknown and it is more likely dilute the positive association between eMRI and LOD. The fact that the association stills persists indicates that it is stronger than it appears. However, we adjusted for injury severity using ICD-9 codes and it would have been good to have objective clinical indicators of severity, which is difficult given pain being a subjective experience.

Another limitation is that we had no data on other significant predictors of work disability duration in occupational LBP, such as worker recovery expectations and fear-avoidance, type of occupation, physical demand of the job, and supervisor support [6, 7, 43, 48]. However, such variables could influence our findings if their distribution differ significantly between states.

## Conclusion

This study found significant cross-state variations in the negative impact of eMRI for occupational LBP on LOD and provided insights into individual and contextual factors associated with these variations. These variations were mainly explained by gender, state WC policy not-limiting initial treating provider choice, higher state orthopedic surgeon density, and lower state MRI facility density. The results suggest that local area characteristics, such as state WC policies and availability of certain types of healthcare play an important role in disability outcomes among workers with occupational LBP who receive eMRI. Targeted healthcare and work disability prevention interventions may improve work disability outcomes in patients with occupational LBP.

## Supplementary information

**Additional file 1: Table S1.** List of ICD-9 Codes for Low Back and Nonspecific Back Injuries or Disorders with code description according to low back injury severity.

**Additional file 2: Table S2.** Sources of census tract level and state level variables, including variable name, level of measurement, source, and link when applicable.

## Data Availability

The datasets used and analysed during the current study are available from the corresponding author on reasonable request with permission of Liberty Mutual, with some restrictions to protect confidentiality of individual data as required by law.

## Abbreviations

BLS: Bureau of Labor Statistics
eMRI: Early magnetic resonance imaging
ICD-9: International Classification of Diseases, Ninth Edition
LBP: Low-back pain
LOD: Length of disability
MHI: Median household income
SAS: Statistical Analysis System
WC: Workers’ compensation
