# Supplementary material for: Why does the adverse effect of inappropriate MRI for LBP vary by geographic location? An exploratory analysis
Source: BMC Musculoskelet Disord. 2019 Nov 30;20:574. doi: 10.1186/s12891-019-2964-7 (PMC6885323; doi:10.1186/s12891-019-2964-7)
Supplement: Supplementary file 1 — Additional file 1: Table S1. List of ICD-9 Codes for Low Back and Nonspecific Back Injuries or Disorders with code description according to low back injury severity. [file 12891_2019_2964_MOESM1_ESM.docx]

**Additional file 1: Table S1.** List of ICD-9 Codes for Low Back and Nonspecific Back Injuries or Disorders

**Format:** DOC

Description: List of ICD-9 Codes for Low Back and Nonspecific Back Injuries or Disorders with code description according to low back injury severity.

| **ICD-9 Code** | **Description** |
| --- | --- |
|  | **More severe low back injuries or disorders** |
| 344.6 | Cauda equine Syndrome |
| 344.60 | Cauda equine Syndrome without neurogenic bladder |
| 344.61 | Cauda equine Syndrome with neurogenic bladder |
| 353.1 | Lesions, lumbosacral plexus |
| 353.4 | Lesions, lumbosacral root NEC |
| 353.8 | Disorder, nerve root/plexus NEC |
| 353.9 | Disorder, nerve root/plexus NOS |
| 355.0 | Lesion, sciatic nerve |
| 721.42 | Spondylosis with myelopathy lumbar region |
| 722.1 | Displacement of thoracic/lumber intervertebral disc without myelopathy |
| 722.10 | Displacement of lumber intervertebral disc without myelopathy |
| 722.73 | Intervertebral dis disorder with myelopathy lumber region |
| 722.83 | Postlaminectomy syndrome of lumber region |
| 724.02 | Spinal stenosis of lumbar region |
| 724.3 | Sciatica |
| 724.4 | Thoracic or lumbosacral neuritis or radiculitis unspecified |
| 724.6 | Disorders of sacrum |
| 738.4 | Acquired Spondylolisthesis |
| 952.2 | Lumbar spinal cord injury without spinal bone injury |
| 952.3 | Sacral spinal cord injury without spinal bone injury |
| 952.4 | Cauda equina spinal cord injury without spinal bone injury |
| 952.8 | Multiple site of spinal cord injury without spinal bone injury |
| 953 | Injury to nerve roots and spinal plexus |
| 953.2 | Injury lumbar nerve root |
| 953.3 | Injury to sacral nerve root |
| 953.5 | Injury to lumbosacral plexus |
| 953.8 | Injury to multiple sites of nerve root and spinal plexus |
| 956.0 | Injury sciatic nerve |
|  | **Less severe low back injuries or disorders** |
| 720.2 | Sacroiliitis NEC |
| 721.3 | Spondylosis, lumbosacral |
| 722.32 | Schmorl’s nodes of lumbar region |
| 722.5 | Degeneration of thoracic or lumbar intervertebral disc |
| 722.52 | Degeneration of lumbar or lumbosacral intervertebral disc |
| 722.93 | Other and unspecified disc disorder of lumbar region |
| 724.2 | Lumbago |
| 724.5 | Backache unspecific |
| 724.7 | Disorders of coccyx |
| 724.70 | Other disorders of coccyx |
| 724.71 | Hypermobility of coccyx |
| 724.79 | Other disorder of coccyx |
| 737.3 | Kyphoscoliosis and scoliosis |
| 737.30 | Scoliosis and Kyphoscoliosis idiopathic |
| 737.39 | Other Kyphoscoliosis and scoliosis |
| 737.4 | Curvature of spine associated with other conditions |
| 737.40 | Unspecific curvature of spine associated with other conditions |
| 737.41 | Kyphosis associated with other conditions |
| 737.42 | Lordosis associated with other conditions |
| 737.43 | Scoliosis associated with other conditions |
| 739.3 | Nonallopathic lesions of lumbar region not elsewhere classified |
| 739.4 | Nonallopathic lesions of sacral region not elsewhere classified |
| 839.2 | Closed dislocation thoracic and lumbar vertebra |
| 839.20 | Closed dislocation lumbar vertebra |
| 939.41 | Closed dislocation coccyx |
| 839.42 | Closed dislocation sacrum |
| 846 | Sprain and strains of sacroiliac region |
| 846.0 | Lumbosacral joint or ligament sprain |
| 846.1 | Sacroiliac ligament sprain |
| 846.2 | Sacrospinatus ligament sprain |
| 846.3 | Sacrotuberous ligament sprain |
| 846.8 | Other specified sites of sacroiliac region sprain |
| 846.9 | Unspecific site of sacroiliac region sprain |
| 847.2 | Lumbar sprain |
| 847.3 | Sacrum sprain |
| 847.4 | Coccyx sprain |
|  | **More severe nonspecific back injuries or disorders** |
| 721 | Spondylosis and allied disorders |
| 721.91 | Spondylosis of unspecific site with myelopathy |
| 722 | Intervertebral disc disorders |
| 722.2 | Displacement of intervertebral disc site unspecific without myelopathy |
| 722.7 | Intervertebral dis disorder with myelopathy |
| 722.70 | Intervertebral dis disorder with myelopathy unspecific region |
| 722.8 | Postlaminectomy syndrome |
| 722.80 | Postlaminectomy syndrome of unspecific region |
| 724.0 | Spinal stenosis other than cervical |
| 724.00 | Spinal stenosis of unspecific region |
| 724.09 | Spinal stenosis of other region |
| 729.2 | Neuralgia/neuritis NOS |
| 952.9 | Unspecific site of spinal cord injury without spinal bone injury |
| 953.1 | Injury to dorsal nerve root |
| 953.9 | Injury to unspecific site of nerve root and spinal plexus |
|  | **Less severe nonspecific back injuries or disorders** |
| 349.9 | Unspecific disorders of nervous system |
| 720.0 | Ankylosing spondylitis and other inflammatory spondylopathies |
| 720.1 | Spinal enthesopathy |
| 720.8 | Other inflammatory spondylopathies |
| 720.81 | Inflammatory spondylopathies in diseases classified elsewhere |
| 720.89 | Other inflammatory spondylopathies |
| 720.9 | Unspecified inflammatory spondylopathy, |
| 721.7 | Traumatic spondylopathy, |
| 721.8 | Other allied disorders of spine |
| 721.9 | Spondylosis of unspecific site |
| 721.90 | Spondylosis of unspecific site without myelopathy |
| 722.3 | Schmorl’s nodes |
| 722.30 | Schmorl’s nodes of unspecific region |
| 722.6 | Degeneration of intervertebral site unspecific |
| 722.9 | Other and unspecified disc disorder |
| 722.90 | Other and unspecified disc disorder of unspecified region |
| 724 | Other and unspecific disorders of back |
| 724.8 | Other symptoms referable to back |
| 724.9 | Other unspecific back disorders |
| 737.8 | Other curvature of spine associated with other conditions |
| 737.9 | Unspecific curvature of spine associated with other conditions |
| 738.5 | Other acquired deformity of back or spine |
| 756.15 | Fusion of spine (vertebra) congenital |
| 799.8 | Other ill-defined conditions |
| 839.4 | Closed dislocation other vertebra |
| 839.40 | Closed dislocation vertebra unspecific site |
| 839.69 | Closed dislocation other location |
| 839.8 | Closed dislocation multiple and ill-defined sites |
| 847 | Sprains and strains of other and unspecified parts of back |
| 847.9 | Sprain of unspecified site of back |
| 848 | Other and ill-defined sprains and strains |
| 848.8 | Other specified sites of sprain and strain |
| 848.9 | Unspecified site of sprain and strain |
| 905 | Late effect of musculoskeletal and connective tissue injuries |
| 905.7 | Late effect of sprain and strain without tendon injury |
| 922.3 | Contusion of back |
| 922.31 | Contusion of back |
| 922.32 | Contusion of buttock |
| 922.8 | Contusion of multiple sites of trunk |
| 922.9 | Contusion of unspecified part of trunk |
| 959.1 | Injury trunk |
| 959.19 | Other and unspecified injury of other sites of trunk |
| 959.8 | Contusion of multiple sites of trunk |
| 959.9 | Contusion of unspecified part of trunk |
