# Supplementary material for: Why does the adverse effect of inappropriate MRI for LBP vary by geographic location? An exploratory analysis
Source: BMC Musculoskelet Disord. 2019 Nov 30;20:574. doi: 10.1186/s12891-019-2964-7 (PMC6885323; doi:10.1186/s12891-019-2964-7)
Supplement: Supplementary file 2 — Additional file 2: Table S2. Sources of census tract level and state level variables, including variable name, level of measurement, source, and link when applicable. [file 12891_2019_2964_MOESM2_ESM.docx]

**Additional file 2: Table S2.**

**Format:** DOC

**Title:** Sources of census tract-level and state-level variables

**Description:**

| **Variable** | **Level** | **Source** | **Link** |
| --- | --- | --- | --- |
| Median household income 2006-2010 | Census tract | ACS through NHGIS | https://www.nhgis.org |
| Annual rate of unemployment 2002-2008 | State | U.S. BLS | [http://www.bls.gov/lau/#data](http://www.bls.gov/lau/%23data) |
| Annual state physician density per 100,000 population 2002-2008 | State | U.S. Census | <http://census.gov/library/publications/time-series/statistical_abstracts.html> |
| State Orthopaedic surgeon density per 100,000 population in 2004 & 2006 | State | AAOS |  |
| State MRI facilities density per 100,1000 population | State | IMV medical Information Division |  |
| Annual state workers’ compensation policy on: waiting period before worker is entitled to WC benefits; waiting and retroactive periods; percentage of total income loss paid based on minimum and maximum wage compensation allowance | State | WCRI & U.S. Chamber of Commerce |  |
| Annual state workers’ compensation cost containment effort: employee limited initial treating physician choice, employee limited treating physician change, mandated utilization review program, and treating physical fee schedule | State | WCRI & U.S. Chamber of Commerce |  |

AAOS, American Academy of Orthopaedic Surgeons; ACS, American Community Survey (published every 5 years during study period); BLS, U.S. Bureau of Labor Statistics; NHGIS, The National Historical Geographic Information System, University of Minnesota; WCRI, Workers’ Compensation Research Institute.
